# Plasma-induced, nitrogen-doped graphene-based aerogels for high-performance supercapacitors

**DOI:** 10.1038/lsa.2016.130

**Published:** 2016-10-07

**Authors:** Xue-Yu Zhang, Shi-Han Sun, Xiao-Juan Sun, Yan-Rong Zhao, Li Chen, Yue Yang, Wei Lü, Da-Bing Li

**Affiliations:** 1 Key Laboratory of Advanced Structural Materials, Ministry of Education & Advanced Institute of Materials Science, Changchun University of Technology, Changchun 130012, China; 2 State Key Laboratory of Luminescence and Applications, Changchun Institute of Optics, Fine Mechanics and Physics, Chinese Academy of Sciences, Changchun 130033, China; 3 School of Basic Sciences & Advanced Institute of Materials Science, Changchun University of Technology, Changchun 130012, China

**Keywords:** energy storage, N-doping, plasma, three-dimension

## Abstract

Commonly used energy storage devices include stacked layers of active materials on two-dimensional sheets, and the limited specific surface area restricts the further development of energy storage. Three-dimensional (3D) structures with high specific surface areas would improve device performance. Herein, we present a novel procedure to fabricate macroscopic, high-quality, nitrogen-doped, 3D graphene/nanoparticle aerogels. The procedure includes vacuum filtration, freeze-drying, and plasma treatment, which can be further expanded for large-scale production of nitrogen-doped, graphene-based aerogels. The behavior of the supercapacitor is investigated using a typical nitrogen-doped graphene/Fe_3_O_4_ nanoparticle 3D structure (NG/Fe_3_O_4_). Compared with 3D graphene/Fe_3_O_4_ structures prepared by the traditional hydrothermal method, the NG/Fe_3_O_4_ supercapacitor prepared by the present method has a 153% improvement in specific capacitance, and there is no obvious decrease in specific capacitance after 1000 cycles. The present work provides a new and facile method to produce large-scale, 3D, graphene-based materials with high specific capacitance for energy storage.

## Introduction

The intrinsic two-dimensional (2D) structure of graphene provides unique physical properties, making it possible to fabricate self-assembled, three-dimensional (3D) architectures^1, 2, 3, 4, 5, 6, 7, 8, 9, 10, 11, 12, 13^. Combining functional nanomaterials with 3D graphene structures would enhance their specific applications. Recently, 3D, graphene-based materials have attracted attention due to their porous structure, which provides a high specific surface area and synergistic effects in the composites^14, 15, 16, 17, 18, 19^. The 3D, graphene-based hybrids are becoming candidates for energy storage, such as in Li ion batteries, hydrogen storage and supercapacitors^20, 21, 22, 23, 24^. Several methods have been reported for fabricating 3D, porous, graphene-based structures, including chemical vapor deposition^25^, growth assisted by a template^26, 27, 28^ and chemical self-assembly^29, 30, 31, 32, 33, 34^. In spite of the significant development for constructing 3D graphene-based structures, these commonly used fabrication methods are generally multistep, hard to control and involve harmful chemical agents. Therefore, further development for preparing high-quality 3D structures is still highly desirable.

In addition, the capacitance of intrinsic graphene is not sufficient for commercial applications but can be improved by N-doping^35,36,37,38,39^. The preparation of N-doped graphene sheets by arc discharge/plasma treatment and chemical vapor deposition (CVD) thermal annealing of graphene oxide (GO) with NH_3_ have been reported^35, 40, 41^. Plasma treatment is an eco-friendly and efficient way to produce N-doped graphene sheets, and several reports have demonstrated N-doping of graphene by plasma^42, 43^. However, these reports address 2D structures and are not aimed at energy storage. A method for fabricating high-quality, N-doped, graphene-based, hybrid, 3D structures does not exist.

In this work, we report a novel method for preparing N-doped, 3D, graphene/Fe_3_O_4_, nanoparticle aerogel (NG/Fe_3_O_4_), which can be expanded for large-scale production of nitrogen-doped, graphene-based aerogel and various active nanomaterials can be incorporated into the 3D hybrid structures. The high-quality NG/Fe_3_O_4_ aerogels are acquired by controllable physical treatment of GO. Compared with the 3D reduced-graphene/Fe_3_O_4_ (RGO/Fe_3_O_4_) prepared by the commonly used hydrothermal method, the present method produces greatly improved porous networks and exhibits significantly enhanced supercapacitor performance. The present work provides a new and facile method to produce high-quality, 3D, graphene-based materials for application in energy storage.

## Materials and methods

### Preparation of GO and Fe_3_O_4_ nanoparticles

A modified Hummers method was used to prepare GO. Fe_3_O_4_ nanoparticles (NPs) were prepared by FeCl_3_·6H_2_O (58 mg) and FeCl_2_·4H_2_O (21.5 mg), which were added to 30 ml deionized water and deoxygenated for 15 min with nitrogen gas. After heating to 80 °C, N_2_H_4_·H_2_O (600 μl, 20 wt%) was injected rapidly and kept stirring for 1 h. The resulting Fe_3_O_4_ NPs were separated from the reaction mixture with a magnet after cooling to room temperature.

### Preparation of NG/Fe_3_O_4_

**Figure 1:**
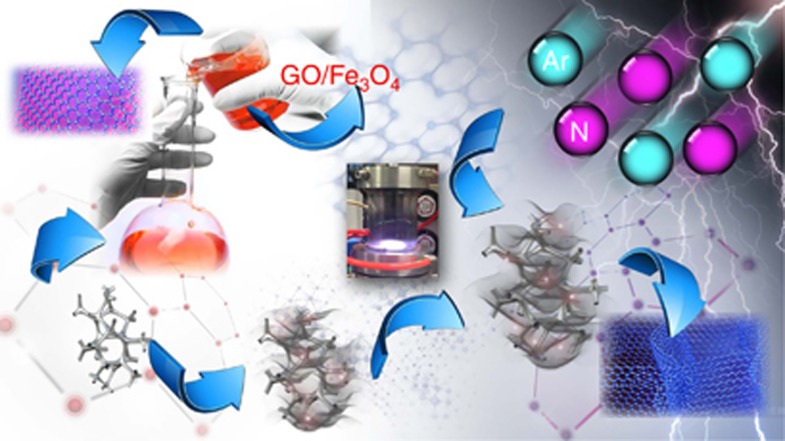
Schematic illustration of the synthetic procedures for NG/Fe_3_O_4_.

A GO solution, including appropriate Fe_3_O_4_ NPs, was stirred for 1 h to obtain the GO/Fe_3_O_4_ suspension. Then, the GO/Fe_3_O_4_ suspension was deposited onto Ni-foam by vacuum filtration, followed by freeze-drying. Finally, the GO/Fe_3_O_4_ composites were reduced and nitrogen-doped simultaneously by hollow cathode (HCD) plasma discharge for 15 min in Ar and N_2_ (Figure 1). The as-prepared sample was denoted as NG/Fe_3_O_4_. A schematic view of the HCD system used for the plasma treatment and the plasma experiment parameters are shown in Supplementary Fig. S1 and Table SI, respectively.

### Hydrothermal synthesis of RGO/Fe_3_O_4_

For comparison, RGO/Fe_3_O_4_ fabricated by the commonly used hydrothermal method was prepared, and the electrochemical behavior was investigated. The GO/Fe_3_O_4_ suspension was kept in an autoclave at 180 °C for 12 h, followed by freeze-drying. A paste, including the active materials (RGO/Fe_3_O_4_), conductive carbon black and polyvinylidene fluoride, was used to prepare test electrodes on Ni-foam.

### Characterization and electrochemical measurements

X-ray diffraction (XRD) measurements were performed with CuKa radiation (D-MAX II A, *λ*=0.15406 nm). A VG ESCALAB MKII (Thermo Scientific, Waltham, MA, USA) was used for the X-ray photoelectron spectroscopy (XPS) investigation. Transmission electron microscopy (TEM) images were acquired by a JEOL2010 (JEOL, Tokyo, Japan). Fourier transform infrared spectroscopy (FTIR) curves were obtained on a VERTEX 70 (Bruker, Ettlingen, Germany). The electrical conductivity of NG/Fe_3_O_4_ and RGO/Fe_3_O_4_ aerogel samples was determined via the four-probe method at room temperature. An IVIUMSTAT (Ivium, Eindhoven, Netherlands) electrochemical workstation was used for the electrochemical investigations, and the electrolyte was 6 M KOH. The galvanostatic charge−discharge was measured under different current densities between −1.0 and 0 V. The cyclic voltammetry (CV) was measured at different scan rates (5, 20, 100 and 200 V s^−1^) between −1.0 and 0 V. The electrochemical impedance spectroscopy was acquired from 100 kHz to 0.01 Hz by applying a signal of 14.14 mV.

## Results and discussion

**Figure 2:**
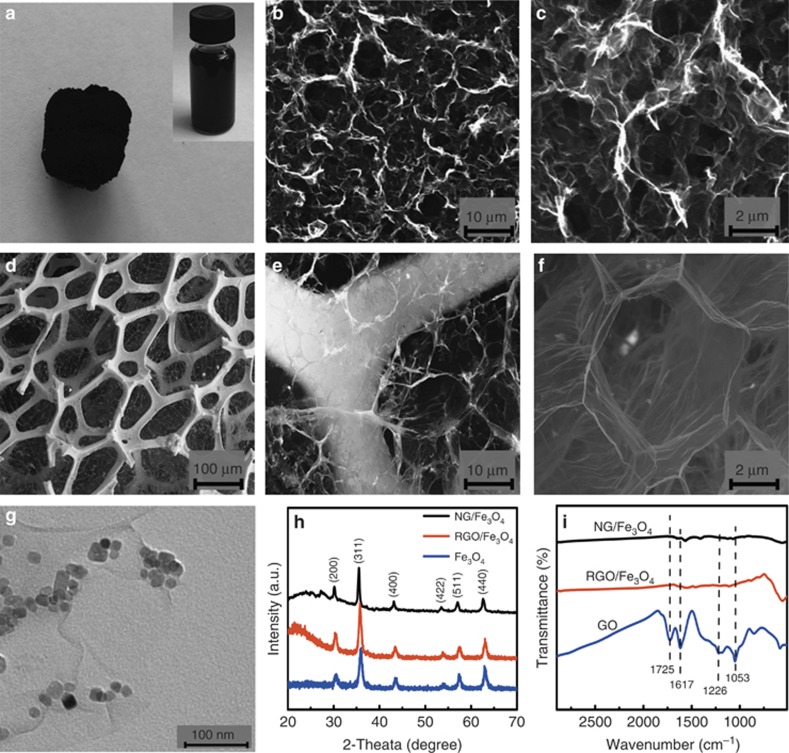
SEM images of RGO/Fe_3_O_4_ and NG/Fe_3_O_4_: (**a**) photograph of hydrothermal 3D RGO/Fe_3_O_4_; (**b**, **c**) SEM images of 3D RGO/Fe_3_O_4_ prepared by the hydrothermal process; (**d–f**) SEM images of 3D NG/Fe_3_O_4_ with different magnifications. (**g**) TEM images of NG/Fe_3_O_4_. (**h**) XRD picture of the as-prepared Fe_3_O_4_, NG/Fe_3_O_4_ and RGO/Fe_3_O_4_. (**i**) FTIR spectra of GO, NG/Fe_3_O_4_ and RGO/Fe_3_O_4_.

Figure 1 shows the experimental procedures of the hydrothermal synthesis of NG/Fe_3_O_4_. The GO/Fe_3_O_4_ mixed suspension was deposited onto Ni-foam by vacuum filtration, followed by freeze-drying. Finally, the GO/Fe_3_O_4_ composites were reduced and nitrogen-doped simultaneously by plasma treatment. Using the hydrothermal method, a gel-like cylinder of RGO/Fe_3_O_4_ was constructed, as shown in Figure 2a. The formation of a 3D porous network with micrometer-sized pores was confirmed by scanning electron microscopy (SEM), as shown in Figure 2b and 2c. However, as in the commonly used hydrothermal methods, the aggregation of graphene sheets during hydrogel formation was inevitable due to the reduction-induced strong π-stacking interaction between graphene sheets, which is originally prohibited by the oxygen-containing surface groups of GO. The network walls of RGO/Fe_3_O_4_ show a tendency of layered aggregation, even though the decoration of Fe_3_O_4_ NPs as spacers on graphene nanosheets partially prevents the aggregation. Compared with hydrothermally prepared RGO/Fe_3_O_4_, NG/Fe_3_O_4_ exhibits greatly improved 3D architectures. The SEM image of NG/Fe_3_O_4_ (Figure 2d) is highly transparent, and the bone of the Ni-foam could be observed clearly. The 3D porous networks are directly formed on the bone by vacuum filtration and freeze-drying. Figure 2e and 2f shows that very thin graphene sheets make up the walls of NG/Fe_3_O_4_. The average size of the pores is several tens of microns, which is larger than that of RGO/Fe_3_O_4_.

For RGO/Fe_3_O_4_ nanostructures, a paste including RGO/Fe_3_O_4_, conductive carbon black and polyvinylidene fluoride was used to prepare test electrodes on Ni-foam. For comparison, a Ni-foam electrode decorated by RGO/Fe_3_O_4_ was directly prepared by the hydrothermal process without further addition of conductive carbon black and polyvinylidene fluoride, as follows: a Ni-foam electrode was soaked in the GO/Fe_3_O_4_ suspension and kept in an autoclave at 180 °C for 12 h, followed by freeze-drying, which resulted in the formation of an RGO/Fe_3_O_4_-decorated, Ni-foam electrode (RGO/Fe_3_O_4_@ Ni-foam) produced by the hydrothermal process, as shown in Supplementary Fig. S2c. The microstructure of the RGO/Fe_3_O_4_@ Ni-foam was similar to that of the RGO/Fe_3_O_4_, as shown in Figure 2a and 2c. In addition, Supplementary Fig. S2d and S2e reveals that the scrolled 3D structures are covered on the Ni bones and have similar morphology to that of RGO/Fe_3_O_4_, and the aggregation of graphene sheets during hydrogel formation was observed. This result indicates that the preparation method of the aerogel plays a key role in avoiding the aggregation tendency in the reduction process. The larger pore sizes and thinner pore walls increased the specific surface area of NG/Fe_3_O_4_ (92 m^2^ g^−1^) compared to that of RGO/Fe_3_O_4_ (55 m^2^ g^−1^) based on the BET results (Supplementary Fig. S3). These properties of NG/Fe_3_O_4_ are directly related to the potential applications from adsorbents to supercapacitors.

Both samples had similar TEM images, and the nanosized Fe_3_O_4_ particles were anchored on graphene uniformly, suggesting efficient assembly between the graphene sheets and the NPs (Supplementary Fig. S4 and Figure 2g). In this work, the diameters of the Fe_3_O_4_ particles were in the range of 10−15 nm. The density and size of the Fe_3_O_4_ particles in the NG/Fe_3_O_4_ and RGO/Fe_3_O_4_ samples were almost identical. Figure 2h shows the XRD curves of Fe_3_O_4_, NG/Fe_3_O_4_ and RGO/Fe_3_O_4_. For all three samples, the main diffraction peaks were assigned to (111), (311), (220), (422), (440), (400) and (511) of the crystal planes of Fe_3_O_4_, consistent with the Fe_3_O_4_ JCPDS card, which suggests that the chemical constitution of Fe_3_O_4_ was retained after the hydrothermal synthesis and plasma treatment. The sharp diffraction peak at 10.3° in GO, as shown in Supplementary Fig. S5, was replaced by a broad peak between 20° and 30°, which results from the (002) reflection of the graphene of NG/Fe_3_O_4_ and RGO/Fe_3_O_4_, indicating that GO was reduced by the hydrothermal and plasma treatment. Figure 2i summarizes the FTIR spectra of GO, NG/Fe_3_O_4_ and RGO/Fe_3_O_4_. GO exhibits typical oxygen-related functional groups. The peaks at 1053 and 1226 cm^−1^ are attributed to the C−O and phenolic C−OH vibrations, whereas that of 1725 cm^−1^ is originated from the C=O vibration^44, 45, 46^. For RGO/Fe_3_O_4_ and NG/Fe_3_O_4_, an Fe–O related peak at 570 cm^−1^ was observed^47^, which indicates a C–O–Fe linkage between the graphene nanosheets and the Fe_3_O_4_ NPs.

**Figure 3:**
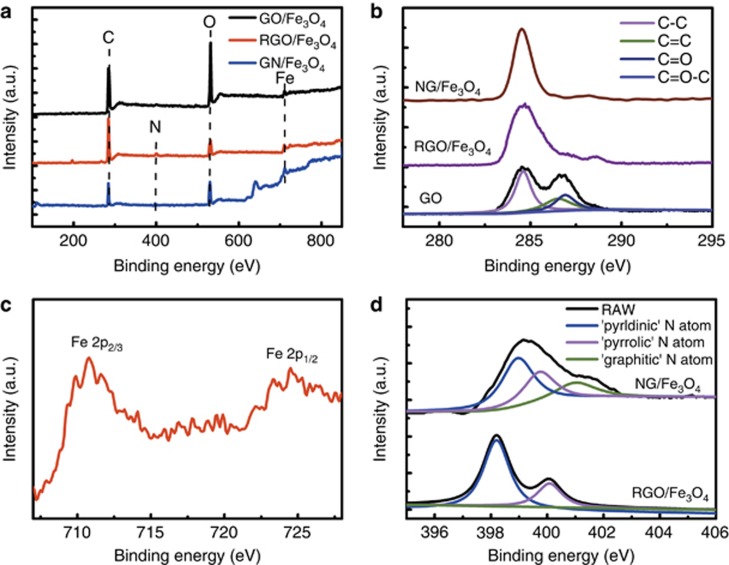
(**a**) XPS surveys of GO/Fe_3_O_4_, RGO/Fe_3_O_4_ and NG/Fe_3_O_4_; (**b**) XPS C 1s spectrum of the GO, RGO/Fe_3_O_4_ and NG/Fe_3_O_4_ samples; (**c**) XPS Fe 2p spectrum of the NG/Fe_3_O_4_ samples; (**d**) XPS N 1s spectrum of the NG/Fe_3_O_4_ and RGO/Fe_3_O_4_ samples.

The XPS surveys of GO/Fe_3_O_4_, RGO/Fe_3_O_4_ and NG/Fe_3_O_4_ are shown in Figure 3a. For all three samples, peaks corresponding to the C 1s and O 1s were observed. Compared with GO/Fe_3_O_4_, the O 1s peak intensities of RGO/Fe_3_O_4_ and NG/Fe_3_O_4_ decreased, suggesting an increased C/O ratio after reduction by the hydrothermal process and plasma treatment, and the oxygen-related functional groups were efficiently removed. This hypothesis was confirmed by the deconvoluted C 1s spectra (Figure 3b). The weak signals of C-O and C=O in RGO/Fe_3_O_4_ compared with that of GO/Fe_3_O_4_ suggest that most of the GO was reduced, and the residual oxygen-related functional groups resulted from the incomplete reduction during the hydrothermal process. However, the oxygen-related peaks in the NG/Fe_3_O_4_ were nearly invisible, which indicates that the plasma treatment was more efficient for the reduction of 3D GO-based hybrids than the hydrothermal method. The formation of Fe_3_O_4_ in RGO/Fe_3_O_4_ and NG/Fe_3_O_4_ was further confirmed by the Fe 2p spectra (Figure 3c). Two characteristic peaks corresponding to Fe 2p_1/2_ and 2p_3/2_ at approximately 724.8 and 711.3 eV were observed, which is consistent with the XRD results. The survey spectra in Figure 3a indicate the presence of nitrogen in both RGO/Fe_3_O_4_ and NG/Fe_3_O_4_. For RGO/Fe_3_O_4_, the introduction of nitrogen is attributed to the reduction agents used in the hydrothermal process, and the nitrogen in NG/Fe_3_O_4_ results from N_2_ plasma treatment. The analysis of the N chemical bonding is shown in Figure 3d, and the N 1s peak can be deconvoluted into three components. The pyridinic and pyrrolic N at 398.2 and 400.1 eV correspond to the N atoms of the π-conjugated system^35, 48, 49^. The graphitic N at 401.7 eV corresponds to the N atoms replacing the C atoms inside graphene sheets, which could be observed clearly for NG/Fe_3_O_4_ but was nearly invisible for RGO/Fe_3_O_4_, as shown in Figure 3d. The first two types of N atoms located in the π-conjugated system account for most of the N in graphene and contribute one or two p-electrons. The graphitic N atoms can be considered to be threefold coordinated sp^2^ N in the hexagonal rings of graphene, which plays an important role in regulating the electronic properties of graphene in electrochemical systems^48, 49^. According to our results, the graphitic N doping is difficult to achieve by the hydrothermal method, and plasma treatment is crucial to achieve a high-quality, N-doping, graphene-based aerogel.

**Figure 4:**
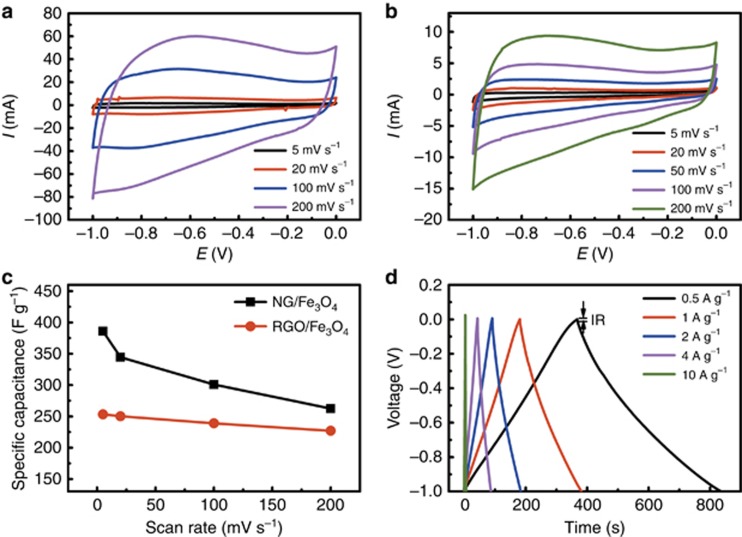
(**a**) CV curves of the RGO/Fe_3_O_4_ electrode. (**b**) CV curves of the NG/Fe_3_O_4_ electrode. (**c**) Variation of the specific capacitance against the scan rate for the RGO/Fe_3_O_4_ and NG/Fe_3_O_4_ electrodes. (**d**) Galvanostatic charge−discharge curves of the NG/Fe_3_O_4_ electrode.

**Figure 5:**
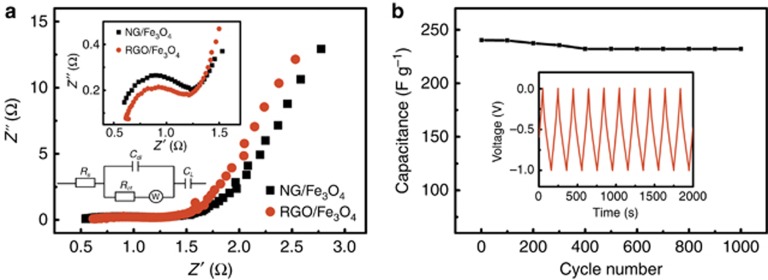
(**a**) EIS of the RGO/Fe_3_O_4_ and NG/Fe_3_O_4_ electrodes, with insets showing the high-frequency parts and the equivalent circuit diagram used for fitting the EIS date. (**b**) Cycle performance of the NG/Fe_3_O_4_ electrode at a current density of 2 A g^−1^. The inset shows the first 10 cycles of galvanostatic charge−discharge. EIS, electrochemical impedance spectra.

A typical three-electrode method was used in this work to investigate the electrochemical behavior. The working electrodes were prepared from RGO/Fe_3_O_4_ and NG/Fe_3_O_4_. The CV curves of RGO/Fe_3_O_4_ and NG/Fe_3_O_4_ are shown in Figure 4a and 4b. The specific capacitances *C* (F g^−1^) can be calculated from the CV curves using the following equation^44^:


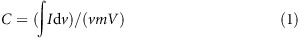


where *V*, *I*, *m* and *v* are the potential window (V), the current (A), the mass of the active materials (g) and the scan rate (mV s^−1^), respectively. Figure 4c summarizes the specific capacitance of the two samples as a function of the scan rate. The NG/Fe_3_O_4_ electrode reached a maximum of 386 F g^−1^ at 5 mV s^−1^, which was much higher than that of the RGO/Fe_3_O_4_ electrode (253.3 F g^−^^1^). Due to the *in situ* preparation of the RGO/Fe_3_O_4_@ Ni-foam electrode, the specific capacitance at 5 mV s^−1^ was 267 F g^−1^, which was slightly improved compared with that of the RGO/Fe_3_O_4_ electrode but was still far behind the NG/Fe_3_O_4_ electrode. The galvanostatic charge−discharge lines of the NG/Fe_3_O_4_ electrode exhibit an almost symmetric triangular shape (Figure 4d), indicating a high reversibility in the charge and discharge cycle^50, 51, 52^. Figure 5a shows the Nyquist plots of the NG/Fe_3_O_4_ and RGO/Fe_3_O_4_ electrodes. For both samples, the Nyquist plots consist of two distinct parts: a linear part at low frequency and a semicircle part at high frequency. The two samples exhibit similar plots. In the high-frequency part (inset of Figure 5a), the charge transfer resistance (Rct) was calculated as 0.9 and 0.85 Ω for the NG/Fe_3_O_4_ and RGO/Fe_3_O_4_ electrodes, respectively. The bulk electrical conductivity of the NG/Fe_3_O_4_ aerogel sample was 174 S m^−1^, three times greater than that of RGO/Fe_3_O_4_ (55 S m^−1^). Although the conductive agent (conductive carbon black in this work) was absent in the preparation progress of the NG/Fe_3_O_4_ electrode, the Rct of the NG/Fe_3_O_4_ electrode has a similar value to that of the RGO/Fe_3_O_4_ electrode, which is also lower than in some previous studies^44, 53^, indicating the excellent conductivity of NG/Fe_3_O_4_.The NG/Fe_3_O_4_ electrode shows excellent cycling stability, as shown in Figure 5b, and there is no obvious decrease in capacitance after 1000 cycles, which is crucial for commercial applications of supercapacitors^54, 55, 56^.

The addition of pseudocapacitor materials is an efficient way to improve the performance of graphene-based supercapacitors. Two very important pseudocapacitor materials are transition metal compounds and conducting polymers. Generally, supercapacitors based on conducting polymers have higher specific capacitance than transition metal compounds; however, their cyclic stability is often poor^57^. Transition metal compounds have improved cyclic stability, but the weaknesses of the transition metal compounds are poor mechanical strength and low electrical conductivity. An efficient strategy to improve supercapacitor performance would be a combination of transition metal compounds in highly conductive 3D graphene frameworks. In the present work, we further developed the commonly used hydrothermal method and have shown that plasma-treated NG/Fe_3_O_4_ would greatly enhance supercapacitor performance. Due to the non-toxicity, easy redox reactions and low cost of Fe_3_O_4_, it has become a good candidate as a pseudocapacitor material, although its theoretical specific capacitance is lower than that of some other transition metal compounds, such as MnO_2_, RuO_2_ and V_2_O_5_^20, 58,59,60^. Table 1 summarizes the performances of supercapacitors prepared with similar 3D systems^20, 58, 59, 61, 62, 63, 64, 65, 66, 67^. For 3D graphene aerogels prepared by CVD with Ni-foam and integrated with oxides^58, 59, 67^, they exhibit large specific surface area and low defects, and these aerogels can be used directly without further reduction. However, the CVD methods generally require rigorous conditions, such as high temperature, templates and dangerous gas. Furthermore, the limited output prevents its expansion for industrialization. In the present work, 3D structures were prepared by *in situ* plasma reduction, which is a simple and feasible strategy that can be expanded for large-scale production of nitrogen-doped, graphene-based aerogel, and various active nanomaterials can be incorporated into the 3D hybrid structures. In addition, the present method demonstrates competitive specific capacitance compared with CVD methods.

Compared with other supercapacitors based on Fe_3_O_4_/RGO structures^61, 62, 63, 64^, the present NG/Fe_3_O_4_ shows excellent specific capacitance and charge transfer ability. Because of the intrinsic properties of the materials, the specific capacitance of the NG/Fe_3_O_4_ electrode is still lower than that of MnO_2_/RGO and RuO_2_/RGO electrodes^20, 58, 59^. However, the present method is a simple and feasible one compared with the traditional hydrothermal process and CVD method. The active nanomaterials can be further expanded for other compounds of transition metals, such as Co, Ni, Mn, Mo and V. The dip-coating and plasma treatment strategy works well for the 3D NG/Fe_3_O_4_ system and would also be effective for other compounds of transition metals. The properties of some other materials, such as cobalt oxide and Ni(OH)_2_, which have ‘battery’ electrochemical behavior, are not compared^68^.

## Conclusions

In conclusion, we have developed a plasma treatment approach to fabricate 3D NG/Fe_3_O_4_ nanostructures as high-performance supercapacitor electrode materials. During the plasma process, the GO of the GO/Fe_3_O_4_ materials was reduced and N-doped. The as-prepared NG/Fe_3_O_4_ electrode exhibited good electrochemical performance, especially high specific capacitance, excellent stability and low charge transfer resistance. As a mature, simple, efficient, low-cost and environmentally friendly method, plasma treatment is a promising process for the preparation and modification of energy storage materials.

## Figures and Tables

**Table 1:** Summary of the capacitive performance of the supercapacitors based on similar structures

**Sample**	**Fabrication method**	***C*s (F g^−1^)**	***R* (Ω)**	***T***	***C* (%)**	**Ref**
NG/Fe_3_O_4_	Freeze-drying/plasma reduction and doping	386	0.9	1000	97	Present work
MnO_2_/RGO	Dip coating	450	6.5	10 000	90	20
MnO_2_/CNT/GE/Ni-foam	CVD	251	1.25	3000	82	58
RuO_2_/CNT/GE/Ni-foam	CVD/dip coating	502.7	1.02	8000	106	59
Fe_3_O_4_/G	2D sandwich-like sheet grown on GO/RGO	349	—	1000	—	61
Fe_3_O_4_/RGO	Hydrogen reducing	262	—	1000	—	62
Fe_3_O_4_/RGO	Hydrothermal	220	1.62	3000	—	63
Fe_3_O_4_/GS	Vacuum filtration/ heat treatment	368	—	1000	—	64
RGO/porous	Electrochemical exfoliation	325	3.6	5000	98	65
3D-RGO	Hydrothermal polymerization/carbonization	225	—	5000	94	66
GE/Ni-foam	CVD	180	0.93	2000	100	67

Abbreviations: *C*, retention rate of *C*_s_ after the cycle life test; *C*_s_, specific capacitance; CNT, carbon nanotube; GE, graphene; GS, graphene sheet; *R*, internal resistance obtained from the electrochemical impedance spectra measurements; *T*, cycles of the cycle life test.
