# Supplementary material for: Plasma-induced, nitrogen-doped graphene-based aerogels for high-performance supercapacitors
Source: Light Sci Appl. 2016 Oct 7;5(10):e16130–. doi: 10.1038/lsa.2016.130 (PMC6059830; doi:10.1038/lsa.2016.130)
Supplement: Supplementary Information [file lsa2016130x1.doc]

Supplementary Information for

Plasma-induced, nitrogen-doped graphene-based aerogels for high-performance supercapacitors

Xue-Yu Zhang, Shi-Han Sun, Xiao-Juan Sun, Yan-Rong Zhao, Li Chen, Yue Yang, Wei Lü, and Da-Bing Li


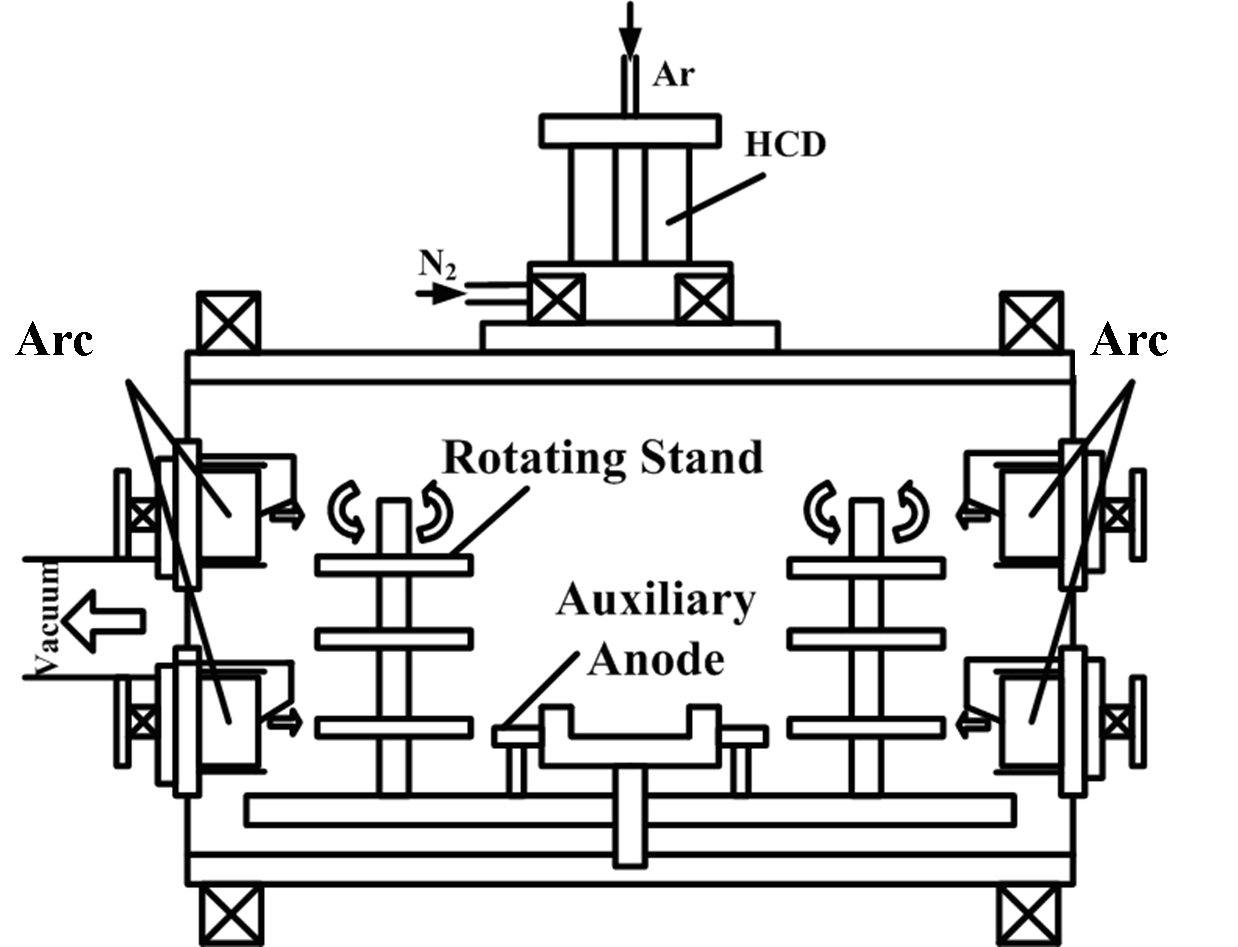


**Figure S1**. Schematic view of the HCD system. The plasma was produced by HCD part (on the top of the system), the sample was put on the rotating stand. In this work, the Arc part was not running.

**Table SI.** Experiment parameters for plasma process

| Parameters | Values |
| --- | --- |
| Ultimate Pressure | 1.0×10^-3^ Pa |
| Working Pressure | 3.0×10^-1^ Pa |
| N_2_ partial pressure | 2.0×10^-1^ Pa |
| Bias voltage | -200 V |
| HCD current | 100 A |
| Cathodic arc current | 80 A |
| Temperature | 〈50 °C |
| Treatment time | 15 min |

For comparison, a Ni-foam electrode decorated by RGO/Fe_3_O_4_ is directly prepared by hydrothermal process without further addition of conductive carbon black and polyvinylidene fluoride (the resulted binder-free sample is defined as RGO/Fe_3_O_4_@Ni-foam). A Ni-foam electrode was soaked in the GO/Fe_3_O_4_ suspension and kept in an autoclave at 180 ^o^C for 12 h followed by freeze-drying in a freeze drier, which results in the formation of a RGO/Fe_3_O_4_ decorated Ni-foam electrode produced by hydrothermal process.


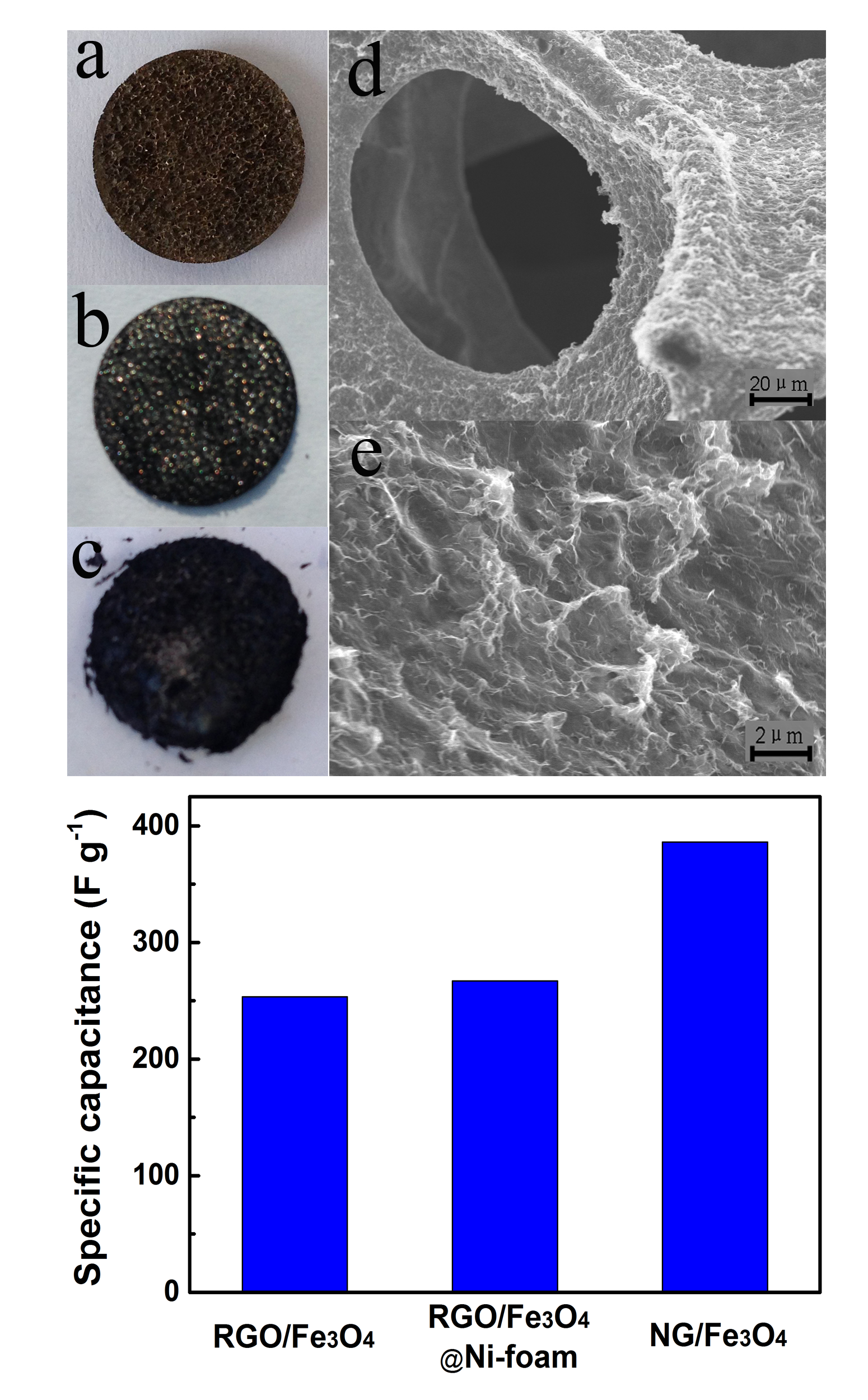


**f**

**Figure S2** Photograph of electrodes (a) NG/Fe_3_O_4_; (b) RGO/Fe_3_O_4_; (c) RGO/Fe_3_O_4_@Ni-foam. (d) and (e) are SEM images of RGO/Fe_3_O_4_@Ni-foam; (f) shows specific capacitance of NG/Fe_3_O_4_, RGO/Fe_3_O_4_ and RGO/Fe_3_O_4_@Ni-foam by CV curves (5 mV s^-1^ ).

The specific (BET) surface area of RGO/Fe_3_O_4_ and NG/Fe_3_O_4_ samples have been measured and shown in Figure S3.


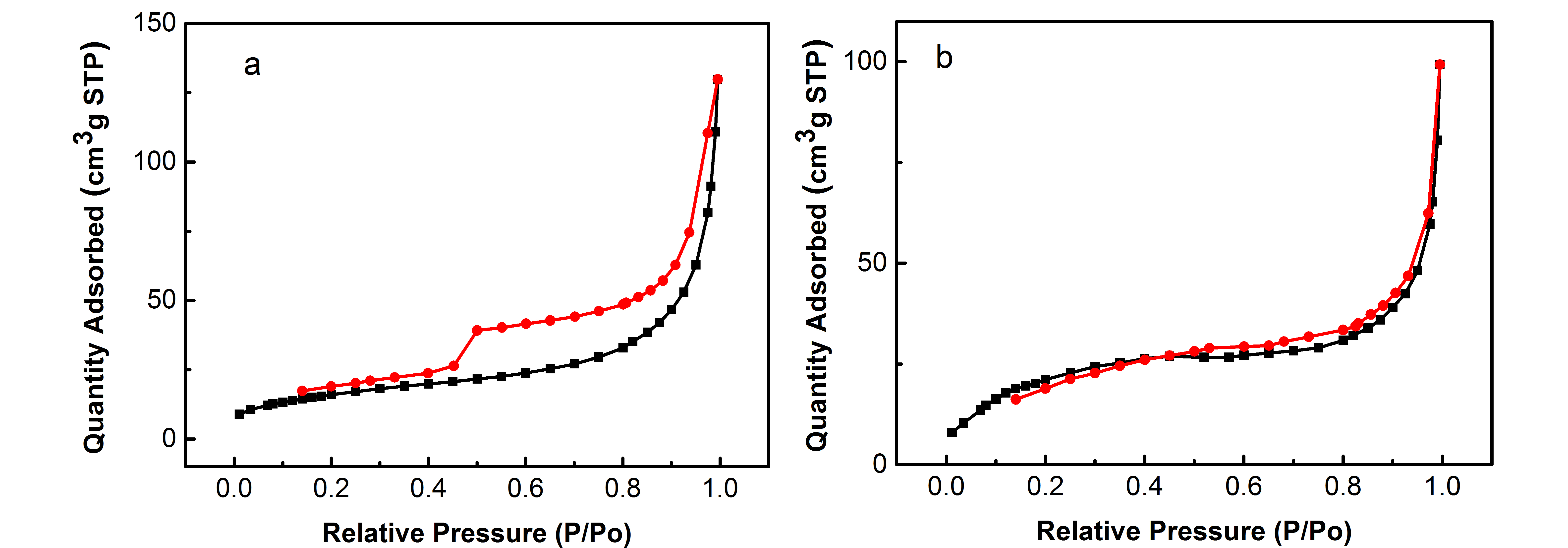


**Figure S3**. Nitrogen adsorption-desorption isotherms of RGO/Fe_3_O_4_ (a) and NG/Fe_3_O_4_ (b).

Figure S4 shows the TEM image of the RGO/Fe_3_O_4_ sample. The Fe_3_O_4_ particles in the range of 10-15 nm were anchored on graphene sheet uniformly.


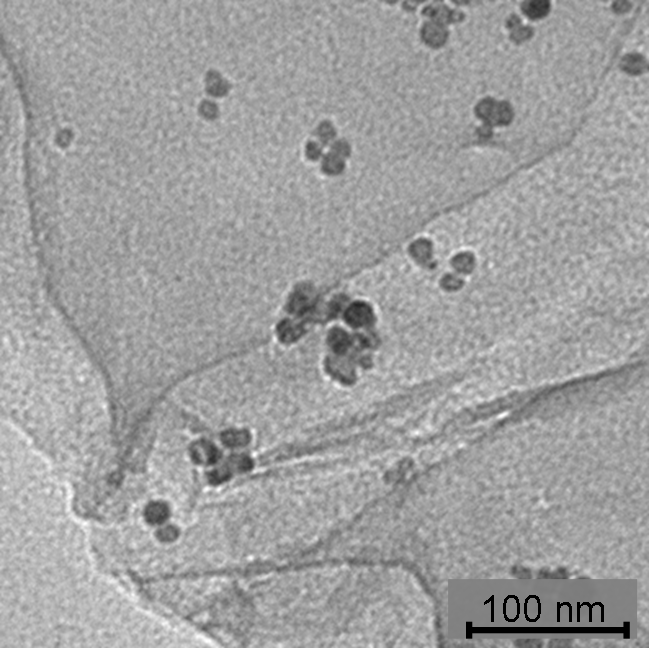


**Figure S4**. TEM images of RGO/Fe_3_O_4_.

The XRD curve of GO is shown in Figure S5. The diffraction peak at 10.3° is a typical feature of GO.


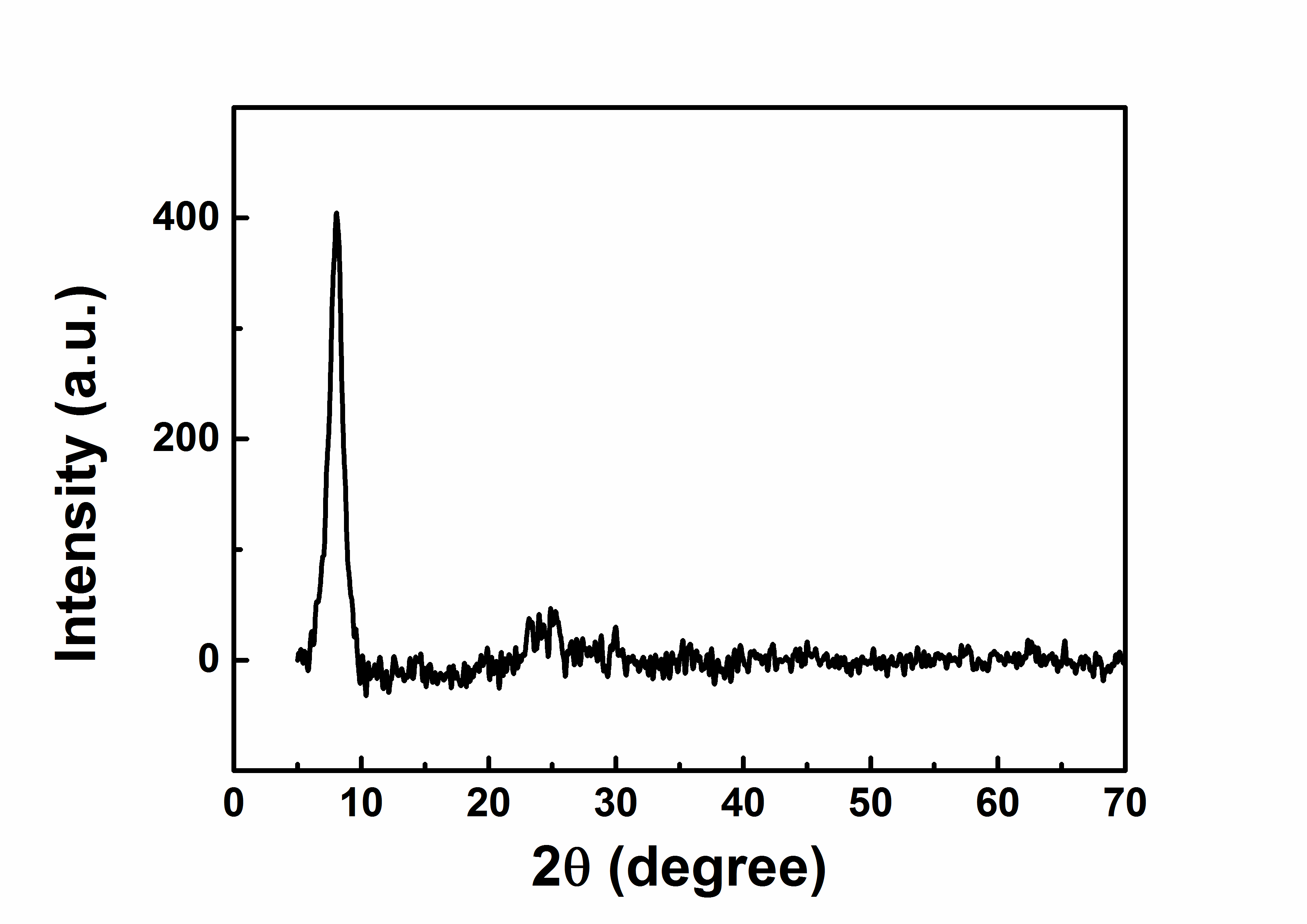


**Figure S5**. XRD picture of GO sample. There is a sharp diffraction peak at 10.3° in the GO sample.

Figure S6 shows magnetization curves of pure Fe_3_O_4_ and NG/Fe_3_O_4_ samples. The value of saturation magnetization of NG/Fe_3_O_4_ samples (62.1 emu g^-1^) is a little smaller than that of pure Fe_3_O_4_ (69.6 emu g^-1^).


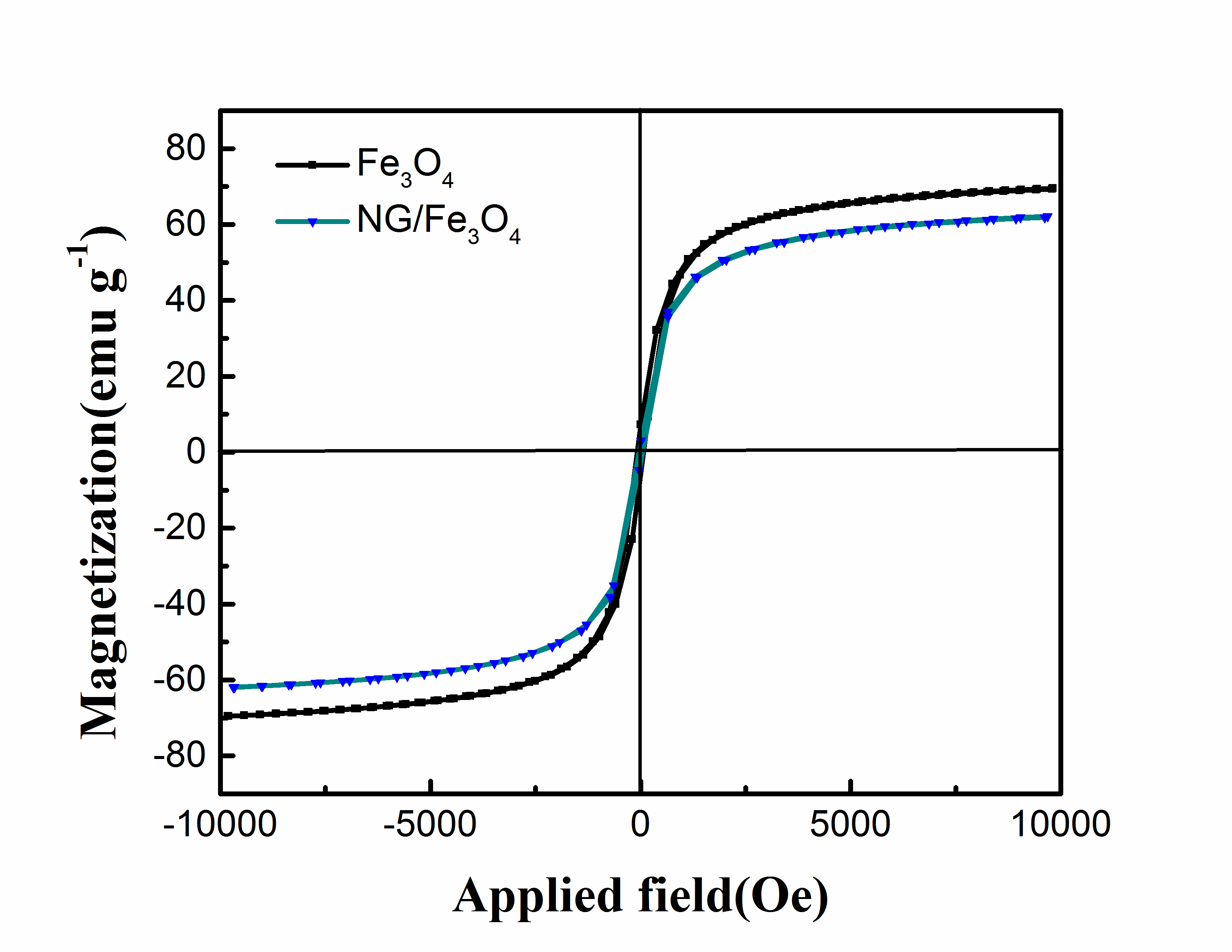


**Figure S6**. Magnetization curves of pure Fe_3_O_4_ and NG/ Fe_3_O_4_ measured at room temperature
